# Splenic Cysts and the Case of Mistaken Identity

**DOI:** 10.7759/cureus.22012

**Published:** 2022-02-08

**Authors:** Rachel M Krzeczowski, Theresa N Jackson, Wareef Kabbani, Heather M Grossman Verner, Phillip Sladek

**Affiliations:** 1 Clinical Research, Methodist Health System, Dallas, USA; 2 Medical Education, Methodist Health System, Dallas, USA; 3 Surgical Pathology, Methodist Health System, Dallas, USA; 4 Surgical Acute Care, Methodist Health System, Dallas, USA

**Keywords:** splenic cysts, general gynecology, endometriosis, splenectomy, trauma

## Abstract

Endometriosis is a well-described pathology, with anatomic location of endometrial cell implantation extending both intraperitoneal and rarely extraperitoneal. Interestingly, previous reports indicated that the spleen enjoys immunity to endometriosis. Here, we present a patient with unremitting abdominal pain who, upon further workup, revealed multicystic disease of the spleen. The patient underwent an open splenectomy with pathology revealing intraparenchymal endometriosis likely due to seeding from traumatic splenorrhaphy. Two-week follow-up demonstrated resolution of symptoms and a well-healing incision with no postoperative complications.

## Introduction

Splenic cysts are a rare entity with only 7.6 cases reported for every 10,000 patients [1]. Fowler et al. provide the current classification system of splenic cysts consisting of either primary (true) cysts with a cellular lining or secondary (false) cysts without a cellular lining [2]. Primary cysts are further subclassified as parasitic versus nonparasitic [2]. Splenic cysts can present as either unilocular or multilocular with or without intramural calcifications [3]. Nonparasitic cysts are typically unilocular, whereas parasitic cysts are more often multilocular [4]. A detailed history and physical examination in combination with computed tomography (CT) can help narrow the etiologic differential diagnoses for patients presenting with splenic cysts.

On the other hand, endometriosis is a fairly common gynecologic pathology with a varied presentation that may present in nearly all intraperitoneal organs [5-7]. Interestingly, past literature indicates that the spleen enjoys a unique immunity against this pathology [8]. Here, we present the first case of a female with endometriosis of the spleen who presented with unrelenting abdominal pain.

## Case presentation

**Figure 1:**
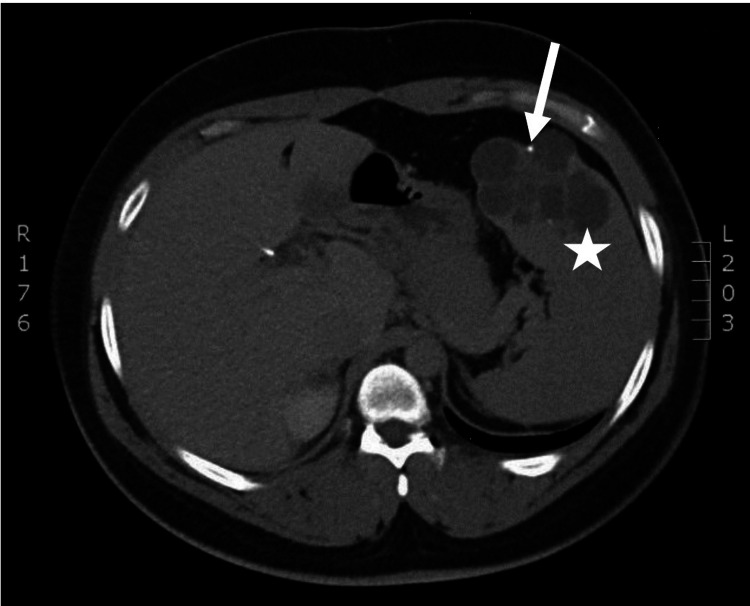
Computer tomography demonstrating a multicystic splenic cyst The spleen contains an 8-cm multiloculated cystic mass (star) with some mural calcifications (arrow) in the cysts.

**Figure 2:**
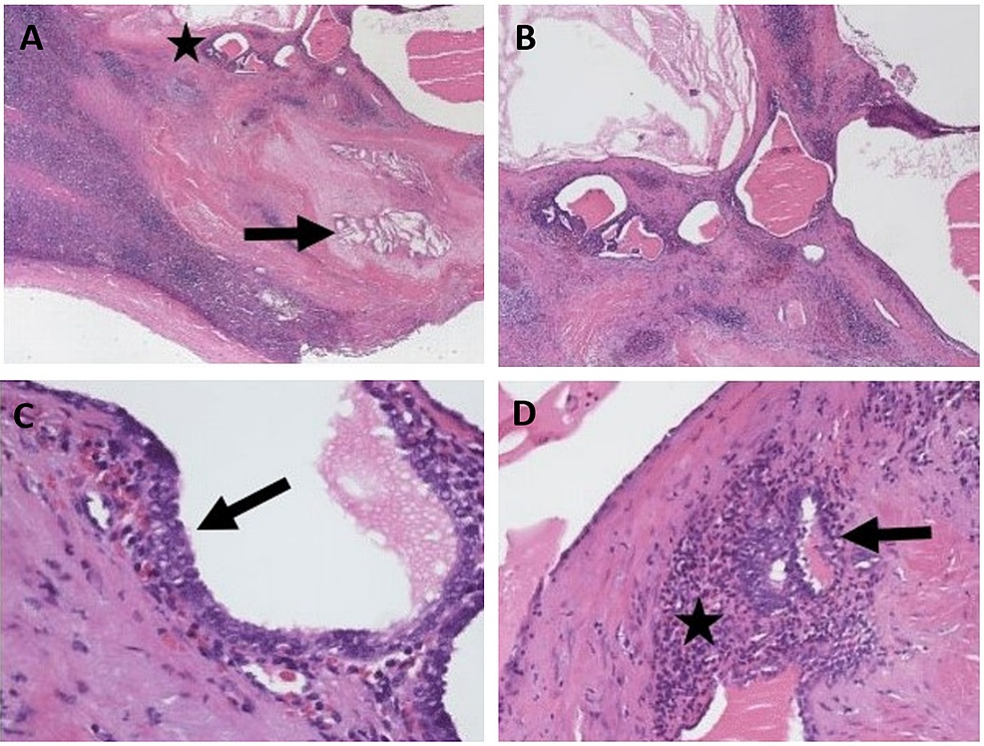
H&E stain of the splenic tissue revealed histology consistent with endometrial cells A: Low-power image showing splenic parenchyma with areas of fibrosis, evidence of past hemorrhage (cholesterol clefts (arrow)) and adjacent cystically dilated glandular structures (star) (H&E, ×20). B: Low-power image showing cystically dilated glandular structures within the spleen. Notice the condensed stroma with hemorrhage surrounding the glands (H&E, ×40). C: Endometrial-type glands with focal ciliated epithelium (arrow) (H&E, ×400). D: Note the endometrial stroma comprised of spindle cells with scant cytoplasm and ill-defined cell borders (star) surrounding endometrial-type glands (arrow) (H&E, ×400).

**Figure 3:**
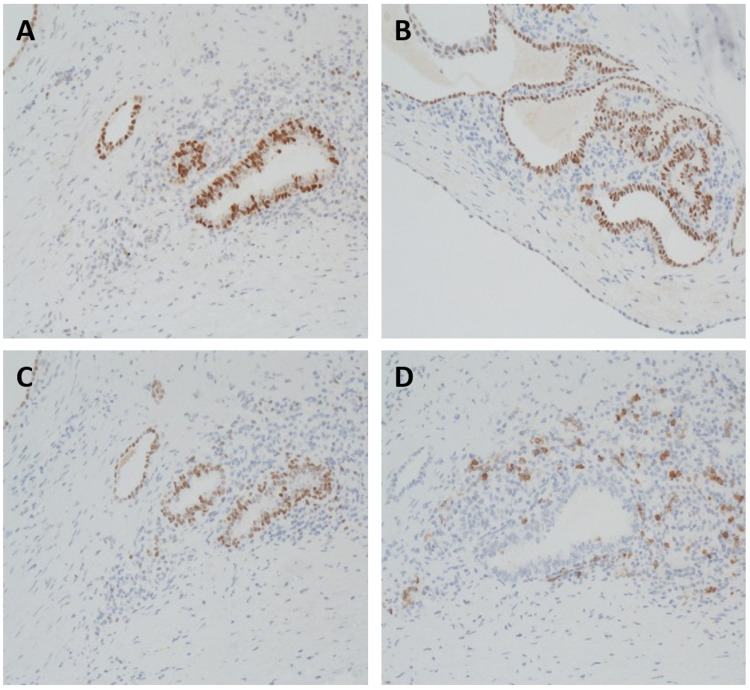
Immunohistochemistry demonstrates normal endometrial tissue with positive staining for Pax-8, WT-1, estrogen receptor, and CD10 Immunohistochemistry defined the endometrial cells with positive Pax-8 (A), WT-1 (B), estrogen receptor staining for epithelial cells of the endometrium (C), and CD10 staining for normal endometrial stroma (D). This is consistent with endometrial tissue.

A 36-year-old female presented with a one-week history of worsening chronic left upper quadrant abdominal pain with postprandial nausea and vomiting. Her medical history was remarkable for splenorrhaphy following blunt traumatic assault 10 years previously. Additionally, the patient had traveled to the Caribbean six months prior to presentation. CT revealed an 8-cm multiloculated cystic mass with mural calcifications (Figure 1). Given her past medical history, the differential diagnosis included both parasitic splenic cyst and pseudocyst. After negative serology for *Echinococcus granulosus* IgG antibodies, the patient underwent an open splenectomy without complications. Gross examination of the resected specimen showed a benign 8-cm multilocular cyst. Microscopic examination revealed multiple cystic structures with glands and surrounding stroma. The glands were lined by mucin secreting columnar epithelium and showed luminal secretions. Stroma was compact and cellular. The tissue underwent H&E staining (Figure 2) and immunohistochemistry (Figure 3), and the findings were consistent with endometriosis. The patient did well postoperatively. At her two-week follow-up, she had resolution of symptoms and a well-healing incision. While continual follow-up was recommended due to the chronic and recurrent nature of endometriosis, our patient was unfortunately lost to follow-up. Follow-up with an annual ultrasound scan of the abdomen for the first few postoperative years would have allowed for recurrence surveillance.

## Discussion

Endometriosis is a common gynecological pathology impacting 6%-10% of reproductive-aged women. Its presentation most commonly includes pelvic pain, dysmenorrhea, dyspareunia, and infertility. Endometriosis is a chronic disease and requires a life-long management plan that can range from medical to surgical therapy. Medical therapy includes nonsteroidal anti-inflammatory drugs (NSAIDs) for endometriosis-related pain, continuous hormonal contraceptives, or gonadotropin-releasing hormone (GnRH) agonists, whereas surgical options include excision or ablation [5].

The pathogenesis of endometriosis has long been debated. The most widely accepted pathogenetic mechanism is the transplantation theory of Sampson, which proposes multiple avenues for seeding of endometriosis, including lymphatic dissemination, vascular dissemination, iatrogenic transplantation, and retrograde menstruation [8]. The most common modality for ectopic endometriosis is overwhelmingly retrograde menstruation, resulting in the implantation of endometrial cells within the pelvic and abdominal cavity [6]. Implantation sites are well documented. Anatomic distribution includes ovaries, fallopian tubes, uterus, sigmoid colon, small bowel, omentum, and other sites [6,7]. Rarely, extraperitoneal endometriosis of the lung, urinary system, peripheral nervous system, and central nervous system have been described [9,10]. It is likely that this patient’s history of splenorrhaphy provided an implantation site for her endometriosis. 

Splenorrhaphy has been reported in surgical literature since the 1800s [11]. However, splenic salvage did not become popularized until the 1970s [12]. By 1980, it was the standard of care for traumatic grade II-IV splenic injuries due to concern for overwhelming post-splenectomy infection and ill-defined postoperative immunologic deficits. The technique itself is diverse and includes topical hemostatic agents, electrocautery, mesh application, partial resection, and omental patching [11]. It is possible that, in this case, omental patching from previous splenorrhaphy provided a splenic implantation site for this patient’s endometriosis. As modern imaging has improved, the landscape of traumatic splenic injury has again shifted to primarily nonoperative management for hemodynamically stable trauma patients, and splenorrhaphy has since been abandoned [12,13]. The change in management for traumatic splenic injury and the paucity of splenic endometriosis suggests that this may be an exclusive case unlikely to be reencountered.

## Conclusions

Our literature review revealed that endometriosis of the spleen has not yet been described. While endometrial splenic implants by retrograde menstruation seem plausible, the unique presentation of intraparenchymal endometriosis implies a multifactorial etiology. Given the patient’s history of splenorrhaphy, we suspect that retrograde menstruation coupled with iatrogenic seeding during splenic repair is the most likely explanation. The current management of traumatic splenic injury has evolved away from splenorrhaphy. Therefore, we believe that this may remain a unique case unlikely to be encountered in the future.

## References

[1] Karfis EA, Roustanis E, Tsimoyiannis EC (2009). Surgical management of nonparasitic splenic cysts. JSLS.

[2] Fowler RH (1921). Surgery of cysts of the spleen. Ann Surg.

[3] Karlo CA, Stolzmann P, Do RK, Alkadhi H (2013). Computed tomography of the spleen: how to interpret the hypodense lesion. Insights Imaging.

[4] McIntyre T, Zenilman ME (2013). Cysts, tumors, and abscesses of the spleen. Current surgical therapy, 11th edition.

[5] Falcone T, Lebovic DI (2011). Clinical management of endometriosis. Obstet Gynecol.

[6] Jenkins S, Olive DL, Haney AF (1986). Endometriosis: pathogenetic implications of the anatomic distribution. Obstet Gynecol.

[7] Cameron IC, Rogers S, Collins MC, Reed MW (1995). Intestinal endometriosis: presentation, investigation, and surgical management. Int J Colorectal Dis.

[8] Rock JA, Markham SM (1992). Pathogenesis of endometriosis. Lancet.

[9] Roberts LM, Redan J, Reich H (2003). Extraperitoneal endometriosis with catamenial pneumothoraces: a review of the literature. JSLS.

[10] Siquara De Sousa AC, Capek S, Amrami KK, Spinner RJ (2015). Neural involvement in endometriosis: review of anatomic distribution and mechanisms. Clin Anat.

[11] Feliciano DV, Spjut-Patrinely V, Burch JM, Mattox KL, Bitondo CG, Cruse-Martocci P, Jordan GL Jr (1990). Splenorrhaphy. The alternative. Ann Surg.

[12] Upadhyaya P (2003). Conservative management of splenic trauma: history and current trends. Pediatr Surg Int.

[13] Stassen NA, Bhullar I, Cheng JD (2012). Selective nonoperative management of blunt splenic injury: an Eastern Association for the Surgery of Trauma practice management guideline. J Trauma Acute Care Surg.

